# Application of Confocal Raman Microscopy for the Characterization of Topical Semisolid Formulations and their Penetration into Human Skin *Ex Vivo*

**DOI:** 10.1007/s11095-022-03245-7

**Published:** 2022-04-11

**Authors:** Nathalie Jung, Sarika Namjoshi, Yousuf Mohammed, Jeffrey E. Grice, Heather A. E. Benson, Sam G. Raney, Michael S. Roberts, Maike Windbergs

**Affiliations:** 1 Institute of Pharmaceutical Technology and Buchmann Institute for Molecular Life Sciences, Goethe University, Frankfurt, Germany; 2 Diamantina Institute, The University of Queensland, Brisbane, Australia; 3 Curtin Medical School, Curtin University, Perth, Australia; 4 Office of Research and Standards, Office of Generic Drugs, U.S. Food and Drug Administration, Silver Spring, MD USA; 5 UniSA Clinical and Health Sciences, University of South Australia, Basil Hetzel Institute for Translational Health Research, Woodville South, Australia

**Keywords:** acyclovir, bioequivalence, confocal Raman microscopy, non-invasive imaging, skin delivery

## Abstract

**Purpose:**

The quality testing and approval procedure for most pharmaceutical products is a streamlined process with standardized procedures for the determination of critical quality attributes. However, the evaluation of semisolid dosage forms for topical drug delivery remains a challenging task. The work presented here highlights confocal Raman microscopy (CRM) as a valuable tool for the characterization of such products.

**Methods:**

CRM, a laser-based method, combining chemically-selective analysis and high resolution imaging, is used for the evaluation of different commercially available topical acyclovir creams.

**Results:**

We show that CRM enables the spatially resolved analysis of microstructural features of semisolid products and provides insights into drug distribution and polymorphic state as well as the composition and arrangement of excipients. Further, we explore how CRM can be used to monitor phase separation and to study skin penetration and the interaction with fresh and cryopreserved excised human skin tissue.

**Conclusion:**

This study presents a comprehensive overview and illustration of how CRM can facilitate several types of key analyses of semisolid topical formulations and of their interaction with their biological target site, illustrating that CRM is a useful tool for research, development as well as for quality testing in the pharmaceutical industry.

**Graphical abstract:**

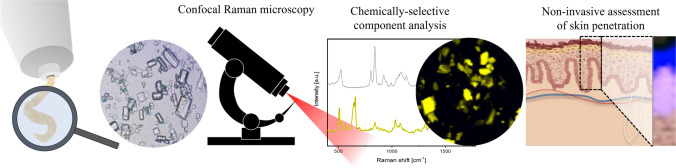

## Introduction

Semisolid dosage forms represent the majority of drug products administered to human skin. Such dosage forms generally contain a combination of different excipients forming a three-dimensional matrix that incorporates the active pharmaceutical ingredient (API or drug) as well as providing specific functions relevant to delivering the drug to the site of action (e.g., modifying skin penetration, potentially by limiting skin penetration for local (topical) therapy or by enhancing penetration for systemic (transdermal) therapy) (1, 2). The intermixing of excipients turns these semisolid dosage forms into a highly complex system with an intricate microstructure that is susceptible to mechanical or thermal strain and associated molecular changes (e.g. polymorphism) during manufacture, storage and/or dose administration, with concomitant changes in composition and/or microstructure. The development of a well-controlled semisolid drug product with reproducible performance characteristics may not only involve controlling the drug concentration, the product stability, and the drug release kinetics, but potentially also the control of dosage form characteristics that can otherwise represent critical failure modes for safety and/or efficacy.

These considerations are relevant to new (innovator) drug products as well as to generic products, which are expected to demonstrate that they are bioequivalent to a reference listed drug (RLD) product (i.e., the innovator, brand name product), or to the currently available reference product. A demonstration of bioequivalence (BE) between a test and reference product traditionally involves evidence that there is no significant difference in the rate and extent to which a drug will become available at the site of action. Pharmacokinetics (PK)-based evidence is often used to support such a demonstration of BE, although other types of evidence comparing the test and reference product can also support a demonstration of BE. The formulation of a generic product can be compared to that of the reference product using three concepts that describe the components, composition, and physicochemical or structural arrangement of matter in the dosage form. The qualitative (Q1) characteristics of a formulation describe its components (ingredients), the quantitative (Q2) characteristics of a formulation describe its composition (formula), and the physicochemical and structural (Q3) characteristics of a formulation describe the manner in which the underlying matter is arranged (3–5).

A demonstration that a proposed generic product is Q1 and Q2 the same as the reference product, and that it also has similar Q3 characteristics, can mitigate the risk of multiple potential failure modes that can alter product performance, and can support a demonstration of bioequivalence for generic products. The Q3 characteristics of a product may be influenced not only by the components and composition of the formulation, but also by the manufacturing process, the storage conditions, dose dispensing, dose administration, or other processes (6). Thus, analytical techniques that can monitor Q3 characteristics are essential to the (comparative) evaluation of such products and should include analyses of the physical state of the active ingredient (crystallinity, polymorphism, etc.), the spatial distribution of the active ingredient of the dosage form, the physicochemical characteristics of the excipients, and the microstructural arrangement of the dosage form, as a whole.

To assess and/or compare the critical quality attributes of a generic and/or reference product, an array of physical and chemical characterization methods may be employed. State-of-the-art analytical tools for the characterization of semisolid topical formulations include Q1 and Q2 characterization as well as tests for Q3 characteristics that can include various rheological analyses to evaluate the deformation of the microstructure in response to stress, thermogravimetric analysis (TGA) to monitor physical and chemical changes as a function of increasing temperature, and differential scanning calorimetry (DSC) for the assessment of temperature-dependent phase transition processes (7–9). A product’s microstructure can also be assessed by imaging techniques ranging from basic light microscopy to scanning electron microscopy, and this can provide information about the existence and size distribution of particle and/or globules, as well as about the overall physical structure of the formulation.

Collectively, the tests that compare the quality and performance characteristics of generic and reference topical products are meaningful because they inform us about the rate and extent to which the drug will become available at the site of action; a demonstration of comparable Q1, Q2 and Q3 characteristics for a prospective generic and reference product mitigates the risks that there could be any significant difference in the rate or extent to which the active ingredient would become available at the site of action, which is the essence of a demonstration of BE (10). The rate and extent to which the active ingredient becomes available in the skin (i.e., the cutaneous PK of the topically administered drug) can be assessed via *in vitro* permeation testing (IVPT) using excised skin mounted in Franz (vertical) diffusion cells or Bronaugh (flow-through) diffusion cells (11, 12). Substances penetrating into and through an excised skin sample (or a polymeric membrane) into an acceptor fluid can be sampled over time and analysed by high performance liquid chromatography (HPLC) for quantitative drug determination. However, information about the spatial distribution of the drug and/or excipients in the skin is practically unfeasible to obtain from IVPT studies and is technically challenging to characterize even by other destructive techniques that may tape strip, heat separate, or dermatome/section the skin (13–15). Skin imaging techniques are better suited to such evaluations. For example, fluorescence microscopy can be used to study the penetration of fluorescently labelled substances into the skin. However, the attachment of a fluorescent label to non-fluorescent molecules of interest may alter their penetration into and distribution inside the skin, and so fluorescence microscopy is not generally suitable for the comparative assessment of a prospective generic product and a commercially available reference product (16–19).

Therefore, based upon the need to better understand and compare the Q3 characteristics of topical semisolid dosage forms, including the physical state and spatial distribution of the drug in the dosage form, as well as the relative amount and spatial distribution of the drug as it becomes available in the skin, Raman spectroscopy microscopy has emerged as an elegant, practical alternative to conventional imaging techniques that can provide critical information to support an assessment of the BE of generic and reference topical products. This laser-based method delivers chemically-selective information with minimal sample preparation effort and its utility has already been established in the fields of geology, crystallography and material sciences (20–22). Due to its accurate chemical selectivity, it is also becoming an increasingly popular pharmaceutical technology for the analysis of polymorphism or to monitor the composition of solid dosage forms like tablets, as well as to support pharmaceutical product design (23, 24). Beyond the analysis of pharmaceutical products alone, Raman spectroscopy has been increasingly implemented on the evaluation of interaction processes between drug molecules and biological tissues. Studies on the penetration abilities of drug molecules as well as excipients so far show promising data on the ability of Raman spectroscopy for penetration analysis (25–27).

The combined application of Raman spectroscopy with confocal microscopy can provide a powerful tool with which to assess and compare critical quality and performance attributes for topical semisolid dosage forms because it combines a chemically selective analysis with a high-resolution non-destructive imaging technique. In this study, we present a comprehensive investigation of different acyclovir creams using confocal Raman microscopy, including multiple versions of a reference product commercially available in the United States (US) the United Kingdom (UK) and Austria, as well as two ‘generic’ products. The acyclovir cream products were analysed with regard to their product compositions, assessing the microstructure of the creams, the polymorphic form of acyclovir in the creams, the spatial distribution of the API and other excipients in the creams, and the penetration behavior of the cream into excised human skin.

## Materials and Methods

### Materials

Micronized acyclovir (polymorphic form V, Fargon, Lot-No. 1311341) was used as a reference material, as well as for the preparation and analysis of the different polymorphic forms of the API. Polymorphic forms I and II were prepared according to Lutker *et al*. (28). In short, polymorphic form I was obtained by heating acyclovir form V to 180 °C (with 5 °C/min) and allowing to cool to room temperature. Polymorphic form II of acyclovir was obtained by mixing 20.0 mg of acyclovir form V in 20 ml of methanol and heating the suspension to 68 °C to dissolve the API. Afterwards the methanol was evaporated quickly. The polymorphic identity was confirmed by comparing the Raman spectra of the prepared polymorphs to spectra published in literature (28). Other reference materials included propylene glycol (Carl Roth, Lot-No. 465235720) and dimethicone 350 (Caelo, Lot-No. 72881487).

#### Topical Products

Three innovator (reference) and two ‘generic’ acyclovir cream products containing 5% acyclovir (w/w) from different manufacturers were evaluated. The innovators (reference products) were Zovirax^®^ (acyclovir) cream, 5% products from US (GlaxoSmithKline, Lot-No. F3002, expiration date: 06/2016), from the United Kingdom (UK, GlaxoSmithKline, Lot-No. C718233 and C711836, expiration date: 03/2018 and 01/2017 for tube and pump products, respectively) and Austria (GlaxoSmithKline, Lot-No. C316, expiration date: 01/2018). The ‘generic’ products were Aciclostad^®^ (STADA, Lot-No. 41863, expiration date: 05/2017) and Aciclovir 1A Pharma^®^ (1A Pharma, Lot-No. EU6728, expiration date: 10/2018) acyclovir cream, 5%, both commercially available in Austria. While most of the products were packaged exclusively in tubes, the Zovirax^®^ UK product was available in a tube as well as in a pump dispenser, and both packaging configurations of this product were studied. All analyses of the products were conducted before the respective expiration dates. Further information on the qualitative and quantitative composition of the products can be obtained via publicly available sources (29–32).

#### Confocal Raman Microscopy

All experiments were carried out using an Alpha300R^+^ confocal Raman microscope from WITec (WITec GmbH, Ulm, Germany) which was coupled to a laser with a wavelength of 785 nm. The laser power was adjusted to 20–50 mW depending on the experimental setup. Analysis of the semisolid products, themselves (not on the skin), was carried out with a lower laser power to minimize agitation of the system by thermal stress, whereas a higher intensity laser power was utilized for the penetration studies into human skin. Using a 50 × objective with a numerical aperture of 0.8, and a pinhole of 100 µm. Raman spectra in a range of 400–1780 cm^−1^ were recorded with a spectral resolution of 4 cm^−1^, lateral resolution of 0.50 µm and axial resolution of 2.05 µm. For the analysis of the topical creams, a sample was evenly spread on a glass slide using a coverslip. Single spectra were recorded by choosing an acquisition time of 10 s (10 accumulations per spectrum). For the acquisition of two-dimensional image scans, bright field images of the formulations were recorded and Raman analysis of the same area was performed. Scans were recorded with a step size of 0.5 µm and an acquisition time of 0.2 s per spectrum.

#### Raman Data Analysis

Processing of all recorded Raman spectra was performed using the ProjectFOUR software (WITec GmbH, Ulm, Germany). Raman spectra were first treated with a cosmic ray removal and a background subtraction using a polynomial fit. To identify the main spectral contributions within the products, the distribution of starting materials and to assess homogeneity of the cream base, a hierarchical cluster analysis (soft k-means algorithm) was applied, where single spectra with similar features are grouped into clusters. To create images based on the spectral information, each cluster was assigned to a specific compound, for example the drug and different excipients were identified based upon their unique Raman spectra. Such clusters comprised an averaged basis spectrum that is characteristic for each compound. Subsequent basis analysis used these basis spectra to fit them to the scanned area, resulting in a color-coded image showing the distribution of the respective basis spectra in the scan region. By combining the images after the fitting, false-colour images were created displaying each compound in a different color.

#### Human Skin Preparation for Penetration Studies

Full thickness human skin was obtained from Caucasian patients undergoing plastic surgery (Department of Plastic and Hand Surgery, Caritaskrankenhaus, Lebach, Germany). Skin donors gave their written consent and the utilization of the tissue in this study was approved by the medical association of Saarland, Germany (application number 204/08). After excision of the tissue, the *stratum corneum* was cleaned with purified water and adipose tissue was removed using a scalpel. The skin tissue was stored in polyethylene bags at -26 °C until further use. For the penetration studies, sections of 25 mm in diameter were punched out of the frozen skin and were allowed to thaw between two filter papers soaked in phosphate-buffered saline (PBS) at room temperature. Afterward, skin samples were cleaned and dried with cotton swaps.

#### Assessment of the Skin Penetration of Semisolid Products

For the penetration studies, a small amount of a semisolid product (approximately 15–20 mg/cm^2^) was applied using circular motions for 30 s onto the clean surface of the skin using a finger covered with a rubber glove. The products were always dosed so that the complete surface of the skin sample was covered. After different time points (4 h and 24 h), the remaining product was removed and the skin surface was carefully cleaned with purified water using cotton swabs and wiped dry afterwards. Raman analysis of skin penetration was performed using the same Raman confocal microscopy setup as described above. The penetration of product into the skin was investigated by recording line scans with a step size of 2 µm from the skin surface downward (in the direction of the z-axis). Each Raman spectrum was treated with a cosmic ray removal procedure and background subtraction was performed, followed by a normalization of the peak associated with the formulation (propylene glycol, 840 cm^−1^) based upon the peak associated with the skin (amide I, ~ 1644 cm^−1^) to account for depth-related attenuation of the Raman signal intensity. The normalized intensity of the peak associated with the formulation was then plotted against the penetration depth to create a penetration profile.

### Assessment of Skin Penetration after Cryosectioning

Human skin tissue was prepared using the same procedure described above for other penetration studies. After incubation of the skin surface with a saturated solution of acyclovir in PG for 24 h, the skin sample was snap-frozen in liquid nitrogen and afterwards cut in 20 µm thick sections using a cryotome (Rotation MEV, SLEE medical GmbH, Mainz, Germany). The skin section was transferred to a glass slide and dried before analysis. To account for z-shifts in focus during Raman measurements, a topographic map was acquired of the sample surface using white light interferometry prior to the acquisition of the Raman scans, using the CRM setup and image acquisition parameters described above.

#### Human Skin Preparation for *In Vitro* Skin Permeation of Acyclovir

Full thickness human skin samples were obtained from patients (female, 26–48 years old) undergoing abdominoplasty at hospitals in Brisbane (Queensland, Austraila) and were refrigerated immediately after surgery. Utilization of the tissue was approved by the Metro South and University of Queensland Human Research Ethics Committees (Approval number: 2008001342) and was carried out in compliance with guidelines of the National Health and Medical Research Council of Australia and FDA RIHSC. Skin samples were further processed by removal of subcutaneous fatty tissue and immersion of full thickness skin in 60 °C water for 60 s, afterwards the epidermal layer was teased off the dermis as described previously (33). Epidermal sheets were air dried and placed in a zip-lock bag for storage at –20 °C until IVPT experiments were performed.

#### *In Vitro* Permeation Test (IVPT) Studies

IVPT studies across human epidermis were carried out in Pyrex glass Franz-type diffusion cells (diffusion area of 1.33 cm^2^; receptor volume of approx. 3.5 ml). Sheets of epidermal tissue were placed between the receptor and donor compartments for equilibration. The receptor solution which consisted of PBS pH 7.4 and 0.01% sodium azide, was stirred with a magnetic stirrer bar. The skin surface temperature was maintained at approximately 32 °C by immersing the receptor compartment in a water bath at 37 ± 0.5 °C. Integrity of the test membrane was assured by measuring the resistance across the epidermis with a digital multi-meter as described before (34). Membranes with an electrical resistance lower than 20 kΩ were rejected from the study. Approximately 15 mg/cm^2^ of pre-weighted amounts of test formulations (acyclovir creams (5%)) were applied to the membranes by spreading evenly with a syringe plunger which was weighed before and after the procedure. Samples of 200 μl samples were withdrawn at various times over a 48-h period from the receptor phase and replaced with equal amounts of fresh pre-warmed receptor medium. API concentrations in the samples were determined via HPLC.

Every test formulation was characterized in IVPT studies by a total of 3 replicates from 6 skin donors. The cumulative amount of API permeated through the epidermis (μg/cm^2^) over time (h) was determined and the flux through the epidermis was calculated and expressed as μg/cm^2^/h.

## Results and Discussion

We studied six different cream products containing acyclovir at the same concentration of 5% (w/w). First, individual Raman spectra were acquired for each cream to identify the components in the creams, with a special focus on acyclovir. As differences in the polymorphic form of a drug in a dosage from can have a significant effect on its therapeutic performance attributes, like its bioavailability (BA) (35), different polymorphic forms of acyclovir were prepared and analyzed as references. Polymorphic form V is the commercially available form that is routinely incorporated into semisolid products for the treatment of *herpes labialis*. Due to their slightly different molecular structure, each polymorph has a characteristic Raman spectrum which allows for a reliable differentiation between these forms.

**Fig. 1:**
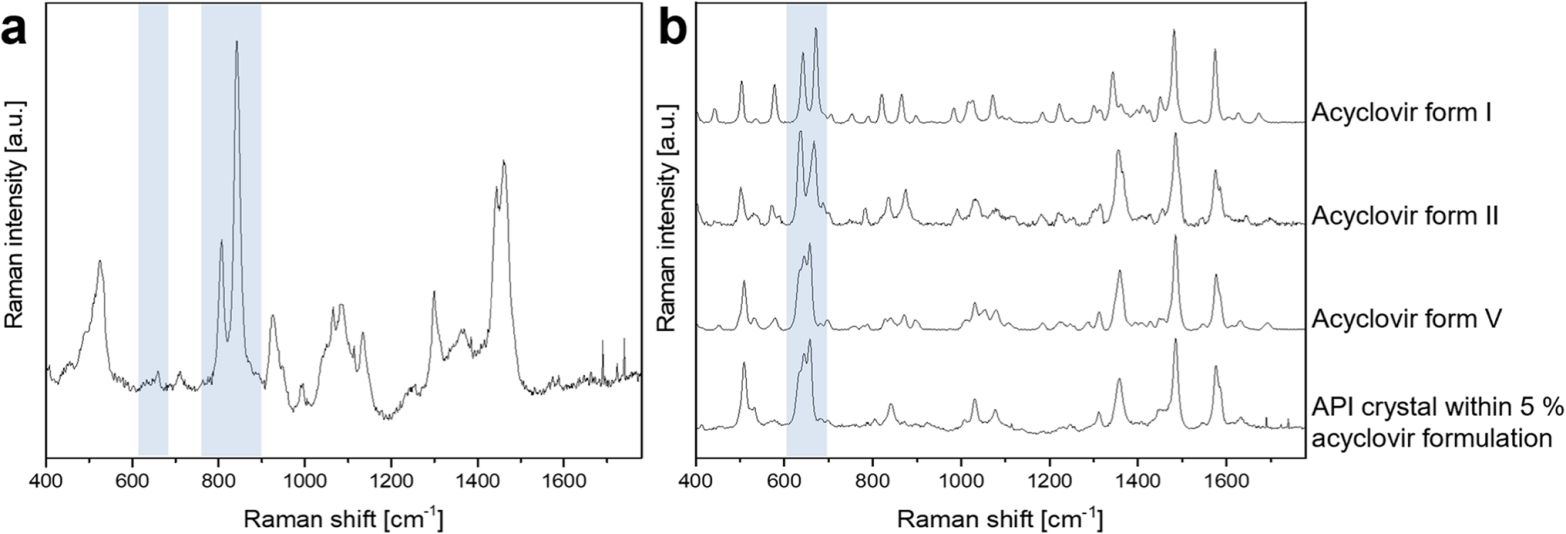
Representative averaged Raman spectrum of an acyclovir cream, 5% product (Zovirax^®^ UK Pump, (**a**) and of polymorphic forms I, II and V of acyclovir as well as of an acyclovir crystal within a commercial cream product (Zovirax^®^ UK Pump, (**b**). The highlighted region marks Raman peaks associated with the putative penetration enhancer propylene glycol (840 cm^−1^) and peaks significant for the distinction of the different polymorphs of acyclovir (650 cm^−1^), respectively.

Figure 1 shows a representative Raman spectrum of an acyclovir cream, 5% (Zovirax^®^ UK Pump) in (a) and a comparison of the individual Raman spectra of different polymorphic forms of the API, acyclovir, in (b). The Raman spectrum of the acyclovir formulation (a) depicts an array of peaks that are attributed to specific molecular bonds of the chemicals in the cream system. Based on the position and shape of the peaks, the ingredients that contribute to the characteristic Raman spectrum of the formulation can be determined. Highlighted in Fig. 1 are peaks associated with the putative penetration enhancer propylene glycol (PG, at 840 cm^−1^) and the API (acyclovir, at 650 cm^−1^). The region around 650 cm^−1^ proved to be highly significant for the differentiation of the polymorphic forms of acyclovir (Fig. 1(b)). Polymorph V shows a distinct peak pattern in this region which can be detected for all analysed acyclovir crystals in the commercial creams. Thus, Raman microscopy could successfully be used to investigate the polymorphic form of acyclovir in the formulation.

**Fig. 2:**
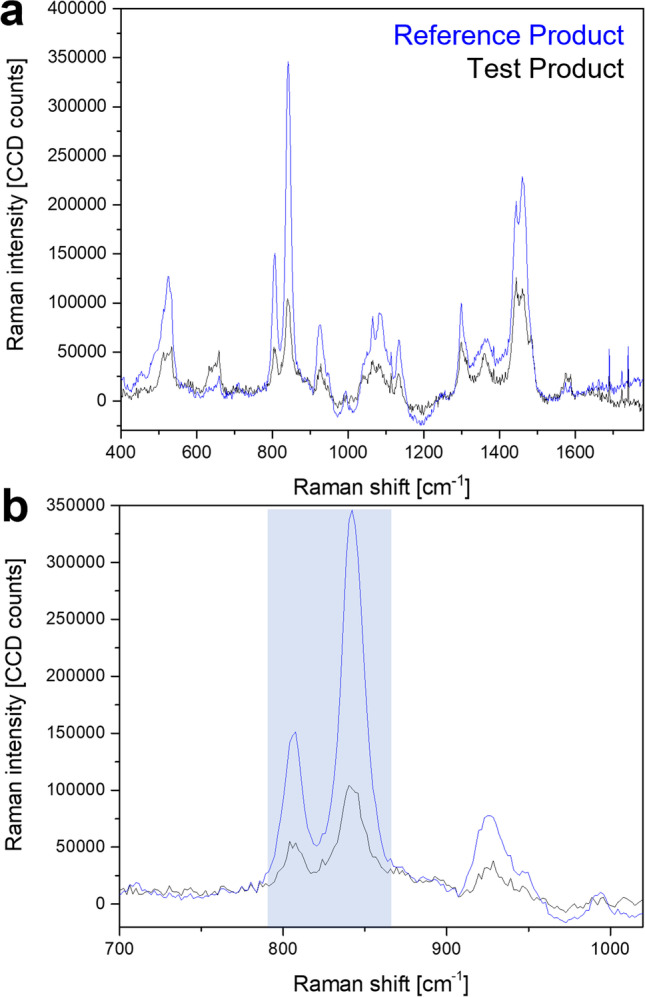
Comparison of Raman spectra from the cream base of a reference product (Zovirax^®^ UK tube) and a ‘generic’ (Aciclostad^®^) acyclovir cream, 5% (**a**). The enlarged section shows specific Raman peaks associated with propylene glycol in the highlighted region (**b**).

As the Raman signal intensity can be linearly correlated with the concentration of the analyte, the technique further allows for the acquisition of quantitative data (36). As an example, the Raman peak associated with PG, which is likely added to the formulations as a putative penetration enhancer, was analysed by Raman microscopy. Comparing averaged Raman spectra from the base of one of the reference products (Zovirax^®^ UK tube) with one of the test products evaluated (Aciclostad^®^), the difference in PG content becomes obvious. The highlighted area in Fig. 2 marks two characteristic peaks that are associated with PG. While the peak at 840 cm^−1^ in the ‘generic’ product reaches an intensity of approximately 100,000 CCD counts, the intensity of PG in the reference product is about 3.5 times as high. Such Raman data can be used for the relative quantification of specific compounds in comparable formulations, and if calibrated to a reference standard, it is possible to quantify specific compounds using this technique. Quantification of PG in both products was independently carried out in another study using thermogravimetric analysis (TGA) which confirmed a similar ratio of PG in these formulations, with approximately 13% of PG in the ‘generic’ product and 42% of PG in the reference product. These findings concur with publicly available information on PG content for reference products (40% PG) and Aciclovir 1A Pharma^®^ (15% PG) (28–31). No information on PG content in Aciclostad could be obtained from publicly available sources. These results suggest that Raman microscopy could be used not only to identify the excipients in a formulation, but also to provide quantitative assessments of the amounts of individual substances, given an accurate calibration of the setup and a consistent measurement procedure.

Optical microscopy can provide insights into the microstructural composition of the formulation and can give information about the shape and size of solid or liquid objects like crystals and globules, if present. However, optical microscopy alone cannot provide information about the chemical composition of the formulation that is being investigated. In this context, confocal Raman microscopy has the advantage that the sample can be investigated optically, and at the same time, a specific position within the imaged sample can be analysed regarding its chemical composition without the need for extensive sample preparation or destruction.

**Fig. 3:**
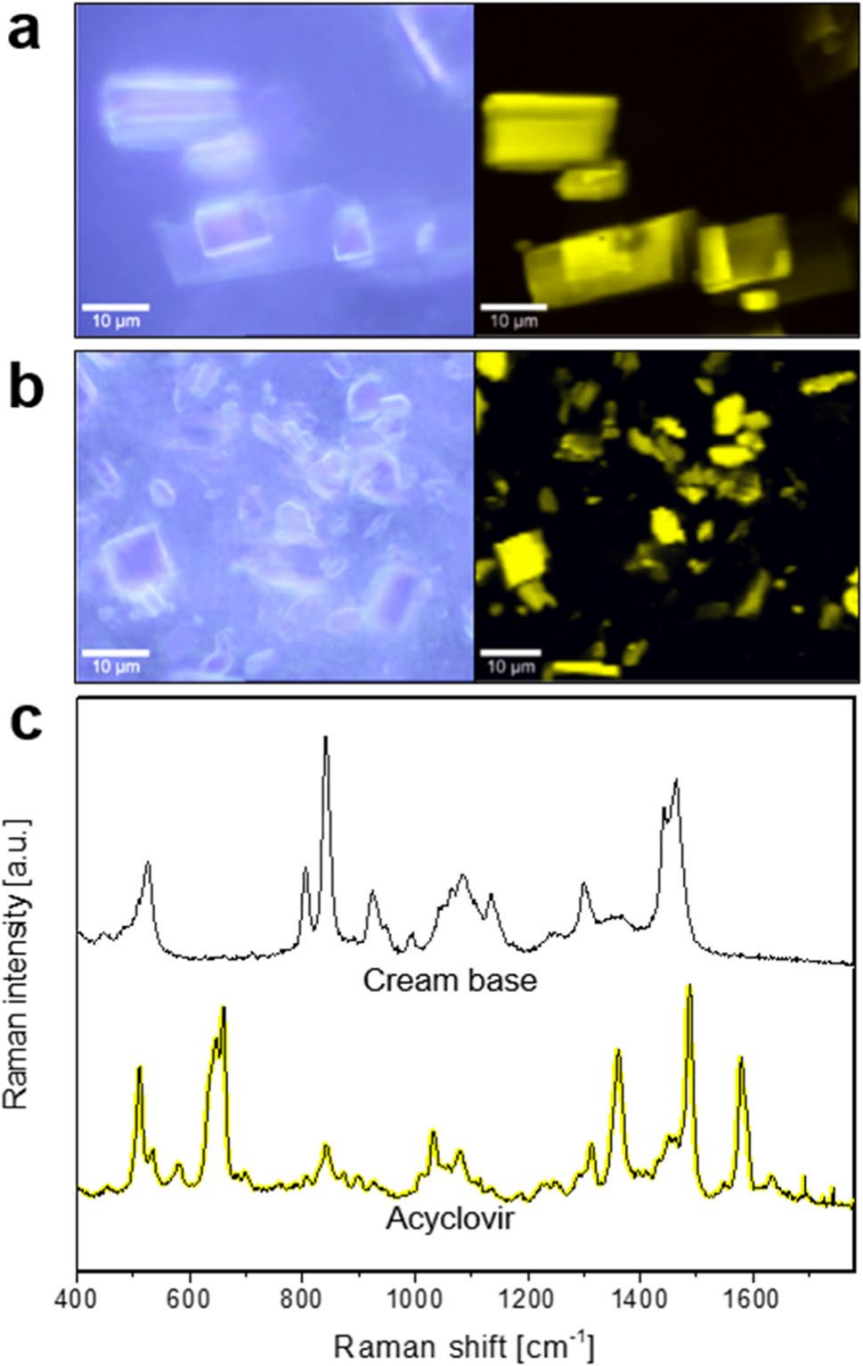
Differentiation of reference (Zovirax^®^ Austria, (**a**) and ‘generic’ (Aciclovir 1A Pharma^®^, (**b**) acyclovir cream, 5% products in bright field (left) and false color Raman (right) images after cluster analysis. Averaged Raman spectra of the clusters shown in (**c**) depict the cream base (black) and drug crystals (acyclovir, yellow). Scale bar: 10 µm.

Figure 3 depicts an example of the combination of optical and Raman microscopy carried out on a reference product (Zovirax^®^ Austria) and a ‘generic’ formulation (Aciclovir 1A Pharma^®^), both containing 5% acyclovir. Bright field microscopy analysis of the reference product (Fig. 3(a)) and the ‘generic’ product (Fig. 3(b)) shows crystals dispersed in a cream base, with large crystals in Fig. 3a and numerous small crystals in Fig. 3b. By using Raman microscopy for the investigation of the same sample area, additional information on the chemical properties is gathered. Applying multivariate data analysis for the interpretation of the acquired Raman data set, regions with similar spectral features are grouped together to form a “cluster” in order to create false colored images for a better visualization of the sample. After thorough analysis of the spectral dataset of the sample shown in Fig. 3, two clusters are revealed; one for the cream base, colored in black, and one for the drug crystals, colored in yellow. In this way, a false colored image is created that shows the API (acyclovir, yellow) embedded in the cream base (black), which can be seen on the right-hand side in Fig. 3(a) and (b). By applying Raman microscopy in addition to light microscopy on the same area the difference in shape (crystal habit) of the drug crystals becomes more apparent, showing sharp-edged rectangular crystals in the reference product, compared to crystals with relatively more irregular and rounded edges in the ‘generic’ formulation.

The (averaged) Raman spectra corresponding to the acyclovir crystals and the cream base of the reference product imaged in Fig. 3a are shown in Fig. 3(c). By comparing these spectra with reference spectra for each of the individual ingredients of the formulation, it is possible to determine the individual contribution of the ingredients to the spectra for the cream base (i.e., the formulation vehicle).

During the acquisition of a Raman scan in the x–y or x–z direction, information from large areas of a sample can be obtained and used for chemical analysis. These area scans are used to investigate the microstructure of the system and to evaluate the homogeneity of the formulation base (vehicle). By choosing a high number of pixels for the area of the sample that is being investigated, Raman spectroscopy can reach a resolution of up to 200 nm, depending on the technical setup. Comparing all the individual Raman spectra that are acquired within the area of the scan, a semisolid system can be investigated for its compositional homogeneity in a way that requires neither extensive sample preparation procedures nor destruction of the sample. Further, this experimental setup allows for the monitoring of compositional changes following the application of mechanical and thermal stress.

The assessment of potential failure modes is a critical aspect of the analysis of pharmaceutical product quality, and the acquisition of a three dimensional Raman scan therefore represents an interesting approach by which to study experimentally simulated product use conditions that can reveal failure modes for product performance; for example, the temperature of the product can be experimentally increased or decreased, and/or mechanical stress can be applied to the product while observing any changes under the microscope. The effect of mechanical stress on an acyclovir cream, 5% formulation (Zovirax^®^ UK pump) was investigated by using a pump (as the container closure system) for dispensing the product. To evaluate the influence of the mechanical stresses of pumping the cream through a nozzle, the same cream was evaluated prior to pumping (cut open from inside the pump canister) as well as after the application of the mechanical stress and shear forces of being pumped out.

**Fig. 4:**
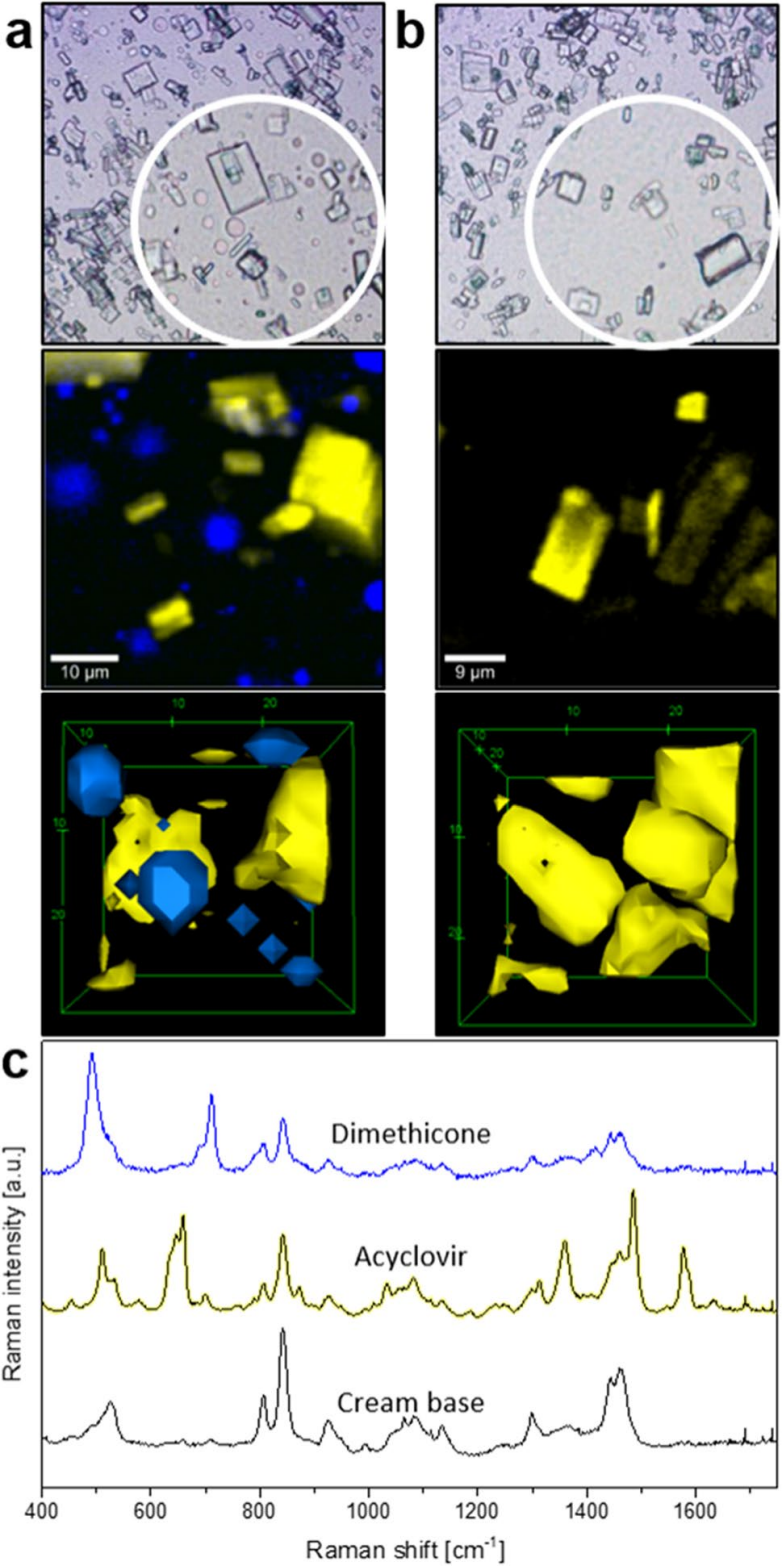
Influence of mechanical stress on product integrity. An acyclovir cream, 5% product (Zovirax^®^ UK pump) was dispensed from a pump container (**a**, scale bar: 10 µm) or obtained by cutting open the pump container and extracting the cream product, applying minimal mechanical stress (**b**, scale bar: 8 µm). Transmitted light images (top) and false color Raman images (below), as well as screen shots of 3D reconstructions are depicted, acyclovir (yellow) and dimethicone (blue) can be seen inside the cream base (black). Corresponding Raman spectra of each false color component are shown in (**c**).

Samples of a commercially available acyclovir cream, 5% product (Zovirax^®^ UK pump) were investigated by light microscopy and with corresponding Raman scans after pumping (a) and before pumping (b). The results are depicted in Fig. 4. Thorough data analysis shows the disintegration of the sample after being dispensed from the pump (Fig. 4(a)), indicated by the appearance of blue, round droplets embedded inside the cream base. However, as seen in Fig. 4b, with the cream sample that was obtained from inside the pump canister (which was not exposed to the mechanical stress of pumping), no spectral differences were evident in the cream base, and the base appeared to be homogeneous. Analysis of the Raman spectra of the (blue) globules observed in Fig. 4(a) revealed that the droplets consisted of dimethicone, a silicone which is incorporated into topical formulations to enhance viscosity and spreadability (37). Despite the benefits of dimethicone for the application of a topical formulation upon the skin, if dimethicone phase separates from the cream base and coats the skin surface, it could form a barrier between the formulation and the skin that could adversely affect the penetration of the API into the skin. This scenario illustrates how the physicochemical and structural arrangement of matter in a semisolid dosage form can influence its therapeutic performance in critical ways, and how the spatially-resolved and chemically-selective information provided by Raman microscopy provides a powerful approach by which to evaluate such potential failure modes for a drug product under experimentally simulated conditions of product use.

The presentation of the false color images seen in Fig. 4(a) and (b) allows for a three-dimensional visualisation of the system when acquiring x–y image scans in multiple z-levels. Corresponding 3D animations to the screen shots in Fig. 4 can be viewed in the electronic supplementary data.

**Fig. 5:**
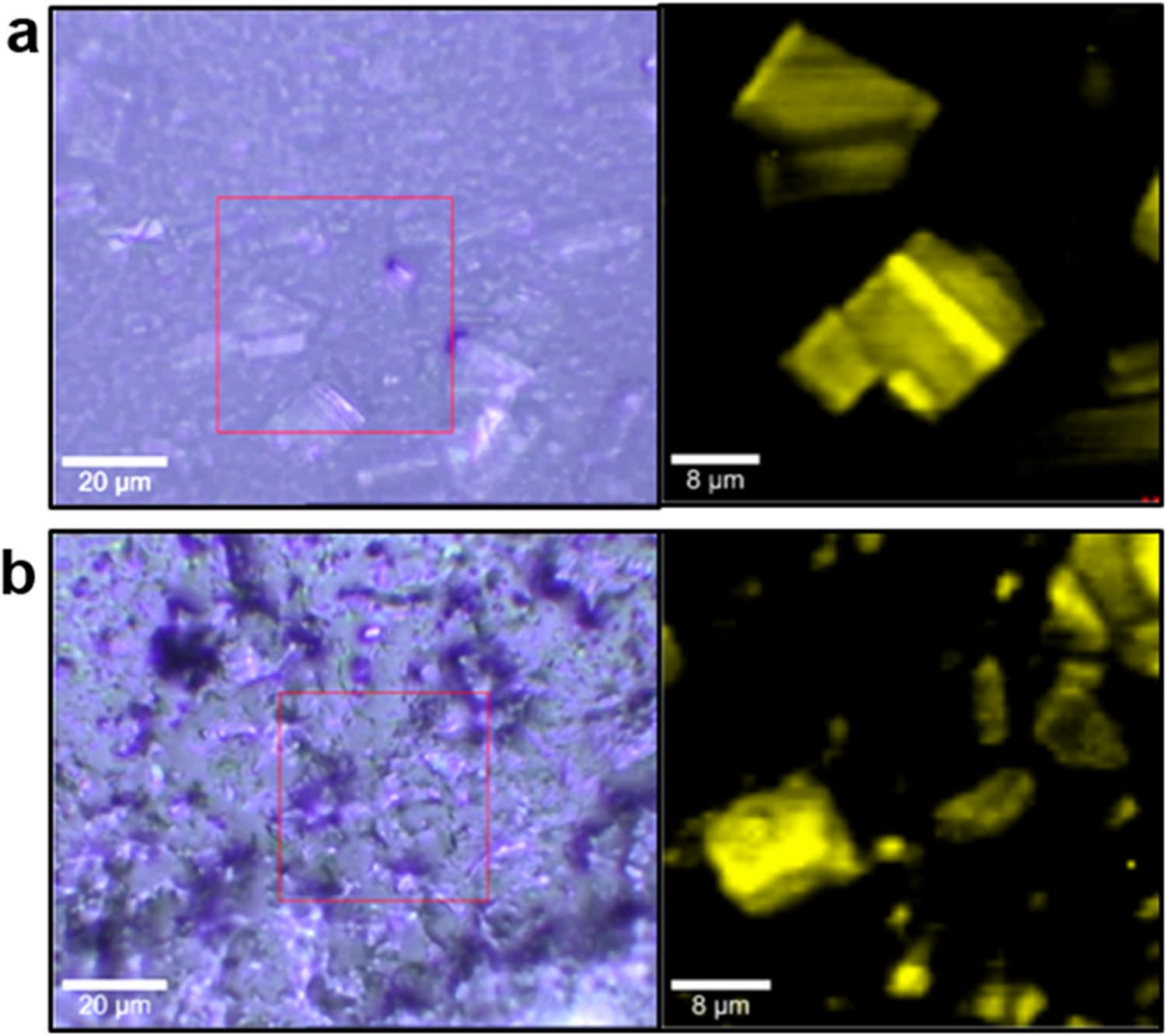
Influence of thermal stress on product integrity. A reference acyclovir cream, 5% product (Zovirax^®^ Austria, (**a**) and a ‘generic’ formulation (Aciclostad, (**b**) were exposed to a temperature of 32 °C for 30 min. Bright field images (left) and false color Raman scans (right) are depicted; after cluster and basis analysis, acyclovir (yellow) can be seen to be embedded in the cream base (black).

Additionally, the influence of thermal stress on a reference (Zovirax^®^ Austria) and a ‘generic’ (Aciclostad) acyclovir cream, 5% product was assessed with confocal Raman microscopy. Figure 5 compares bright field images and corresponding Raman scans of these two products after equilibration to 32 °C (*in vivo* human skin temperature) for 30 min. The bright field microscopic images show drying-related changes in the optical appearance of the creams, but the Raman scans show no apparent changes in chemical composition compared to unstressed samples. In both the reference (a) and the ‘generic’ (b) product, the drug crystals (yellow) are clearly visible in the cream base (black) and show similar morphological properties to those seen in the unstressed products. Following the thermal stress, no disintegration of the cream base could be observed in either the reference, or the ‘generic’ product. Furthermore, no differences in Raman spectra between the API before and after application of thermal stress could be observed, which demonstrates the likely stability of these products during product use.

Thus far, we have discussed the utility of Raman spectroscopy and microscopy to characterize potentially critical quality attributes of topical semisolid drug products, focusing on characterizing the dosage form itself. However, Raman microscopy also has the potential to characterize and compare the performance of prospective generic topical products compared to their reference product, by characterizing the topical BA and BE of drugs as they penetrate into the skin from a topical product. The cutaneous PK (BA and BE) of topical formulations has conventionally been characterized by an *in vitro* permeation test (IVPT) using excised human skin mounted in diffusion cells, where the rate and extent to which an API permeates into and through the skin is determined (38, 39). Confocal Raman microscopy (CRM) can also be utilized to evaluate the cutaneous PK (BA and BE) of topical formulations by monitoring the penetration of compounds into the uppermost (epidermal) layers of the skin. Some advantages of using CRM to characterize epidermal PK are that it can simultaneously monitor the penetration of the API as well as formulation excipients, at multiple depths within the epidermis, and do so in a non-destructive, non-invasive manner.

After the application of a topical formulation or product upon excised human skin, different acquisition modes can be applied to the sample in order to investigate the interaction of the formulation components with the tissues at the skin surface, and in deeper skin layers. Using stacked virtual cross sections of the skin in the x–z direction, and after various data analysis steps as well as the creation of a false color images, CRM can enable the visualization of the presence of the API as well as other formulation components at different depths in the skin. Alternatively, the acquisition of single line scans (acquisition of individual spectra along the z-axis from the skin surface downwards) can provide a faster, semi-quantitative analysis of the penetration of the API as well as other formulation components to different depths in the skin, which could then be characterized at successive points in time to monitor how the amount of API (for example) changes over time, thereby characterizing the rate and extent to which the API (or other formulation components) becomes available at a particular depth in the skin. Such cutaneous PK profiles could be characterized at each of multiple depths in the epidermis and compared between topical products to evaluate relative BA and BE.

**Fig. 6:**
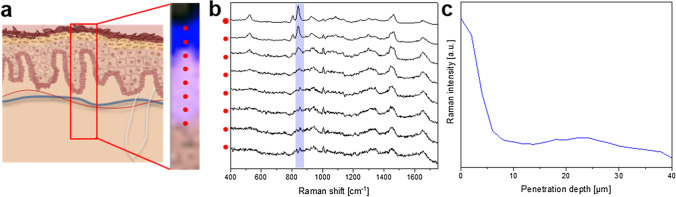
(**a**) Schematic representation of the acquisition of a virtual cross section using a confocal Raman microscope, visualizing skin that was previously incubated with an acyclovir cream, 5% product (Zovirax^®^ US) for 24 h. Following cluster and basis analysis, the resulting image (shown on the right side of (**a**)) indicates the area above the skin sample in black, the PG in the skin in blue, and the skin, itself, in pink; the red dots in the image mark the depths at which single spectra were acquired in in the z-direction, with the corresponding red dots and individual spectra shown in (**b**). The intensity of the Raman peak associated with PG (highlighted in the blue band across the spectra of (**b**)) is plotted as a function of penetration depth in (**c**), producing a profile of the relative amount of PG that is present at different depths in the skin 24 h following the topical administration of an acyclovir cream, 5% product (Zovirax^®^ US).

Figure 6 illustrates the methods of acquisition as well as representative data for the penetration of the excipient PG (monitored by its Raman signal) from an acyclovir cream, 5% product into excised human skin. Figure 6(a) schematically depicts the acquisition of an image scan with CRM in the x–z direction, resembling a cross section of the skin tissue, performed virtually without destruction of the sample. Following several steps of spectral analysis, a false colour image is created where the skin is shown in pink, and the areas where the signal of PG can be detected are depicted in blue (black indicates the area above the skin sample). The acquisition of a virtual cross section, resulting in an image as depicted in Fig. 6(a), gives an illustrative overview of the substance penetrating into the skin tissue. However, the acquisition of such images can be very time consuming, requiring up to several hours, depending on the image size and resolution. This poses a technical challenge that limits how frequently one can acquire repeated (successive) scans to monitor the penetration kinetics at different points in time. For the semi-quantitative analysis of penetration via CRM it is therefore advisable to opt for the acquisition of a series of single spectra, starting at the skin surface and going downward (positions are indicated by the red dots in Fig. 6(a). The PG-related peak of each single spectrum, which is highlighted in blue across the cascaded spectra of Fig. 6(b), is normalised to a skin-derived peak to compensate for depth-dependent signal attenuation. By plotting the normalised intensity of PG against the penetration depth, a profile can be generated illustrating the relative amount of PG at different depths in the skin at a selected point in time, as depicted in Fig. 6(c). The selection of the skin-associated peak for the signal normalization procedure is a crucial step in the process and should be selected taking into consideration the spectral features of the compound whose penetration into the skin is being monitored, as well as the suitability of the skin-associated peak, itself, for the analysis. This approach has been extensively discussed by Franzen *et al*. (40).

**Fig. 7:**
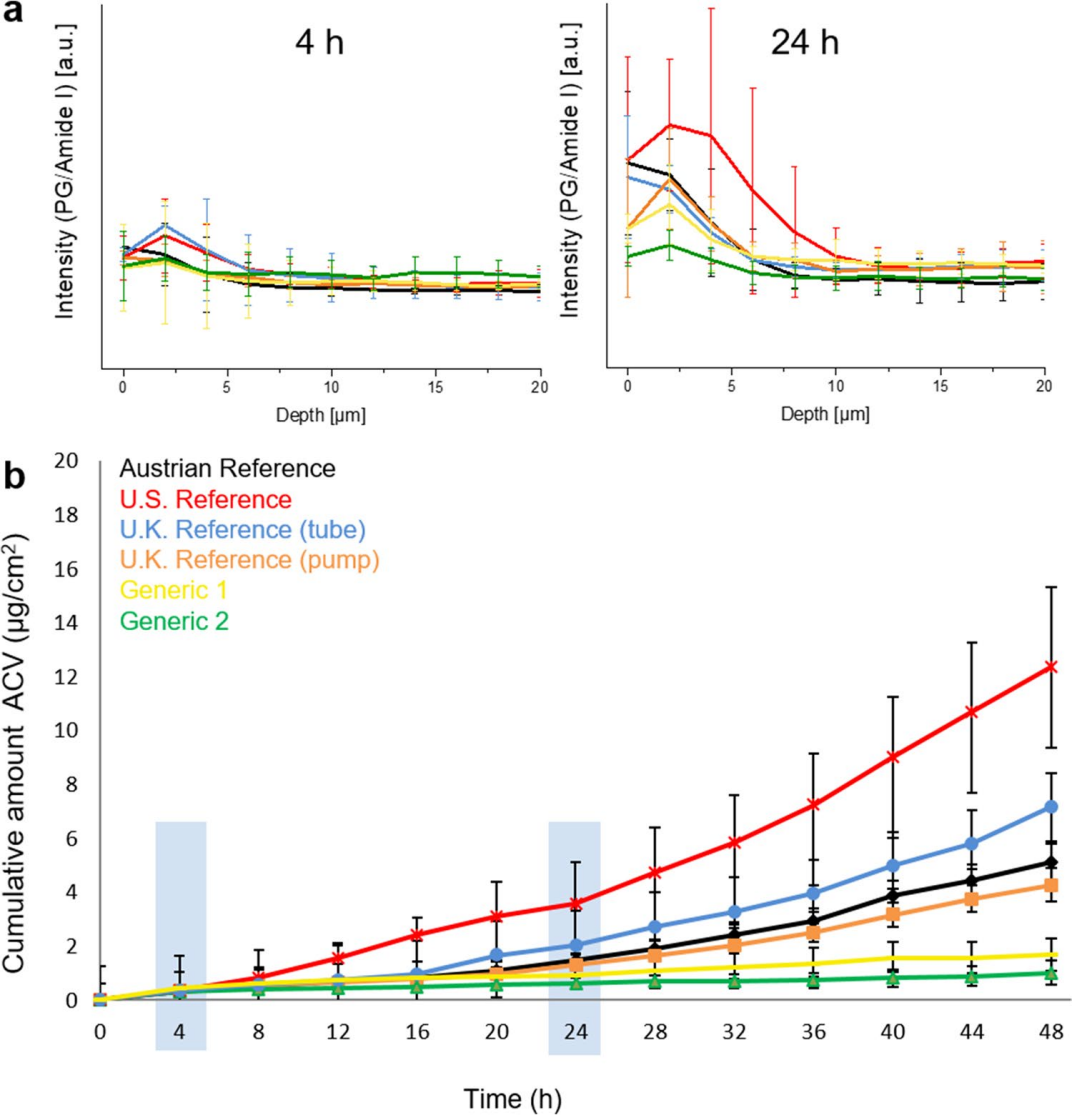
(**a**) Assessment of the penetration of PG into the epidermis of excised human skin from six acyclovir cream by CRM, 5% products, and (**b**) corresponding assessment by an IVPT study of the permeation of acyclovir across the epidermis of excised human skin from the same set of six acyclovir cream, 5% products as were characterized in (**a**).

To illustrate the utility of CRM to compare the cutaneous PK of topically penetrating compounds between different formulations or products, the penetration of six commercial acyclovir topical products was assessed by measuring the intensity of a PG-associated peak, as described above. Two time points (4 h and 24 h) were investigated by CRM and the resulting penetration profiles for all the acyclovir products are compared in Fig. 7(a). At 4 h following the application of the topical products upon the skin, the PG depth-profiles of the six acyclovir products show no significant differences between each other. However, by 24 h after dose application, most of the products exhibit a greater intensity for the PG peak to a greater depth, indicating that a larger amount of PG has penetrated into the skin, and that it has also penetrated deeper. This illustrates the ability of CRM to monitor changes in the amount of a topically penetrating compound over time, and to acquire this information at each of multiple depths in the uppermost layers of the skin. While one acyclovir cream, 5% product (Zovirax^®^ US), shown in the red, exhibits higher PG intensities than the other products in the outermost skin layer (to a depth of approximately 10 µm), and while the various reference products tend to exhibit greater penetration of PG than the ‘generic’ products, the results illustrate more generally how the penetration kinetics of a given compound in a topical product can be compared and differentiated among products. Also, while only two time points were evaluated in this study (4 h and 24 h), these results demonstrate that it is feasible to utilize CRM to monitor changes in the penetration kinetics at a selected depth in the upper epidermis as a function of time, which can thereby produce cutaneous PK profiles that can be compared between topical products. As a further demonstration of the utility of CRM for this purpose, the cutaneous PK results characterized by CRM at 4 h and 24 h were compared to cutaneous PK results for the same topical products evaluated in an IVPT study performed in an independent laboratory (Fig. 7(b)). While the CRM results monitored the penetration of PG instead of acyclovir (because the Raman signal of the dissolved acyclovir in the skin was too weak to utilize), the cutaneous PK results for the different products measured by CRM correlated well with the cutaneous PK of acyclovir from the same set of products measured in the IVPT study. Vertical blue bands at 4 h and 24 h in Fig. 7(b) highlight the cumulative amount of acyclovir that permeated through the skin from each of the topical products in the IVPT study. In a manner precisely corresponding with the CRM results, at 4 h following the application of the topical products upon the skin, the IVPT profiles of the six acyclovir products show no significant differences among each other. However, by 24 h after dose application, the IVPT results show that most of the products exhibit a greater amount of acyclovir penetration into the skin, consistent with the penetration kinetics characterized by CRM. Also, consistent with the CRM results, the IVPT results indicate that the reference products generally exhibit greater penetration than the generics. The IVPT results also show that one acyclovir cream, 5% product (Zovirax^®^ US), shown in the red, exhibits substantially greater penetration than the other products, confirming the results obtained from CRM.

**Fig. 8:**
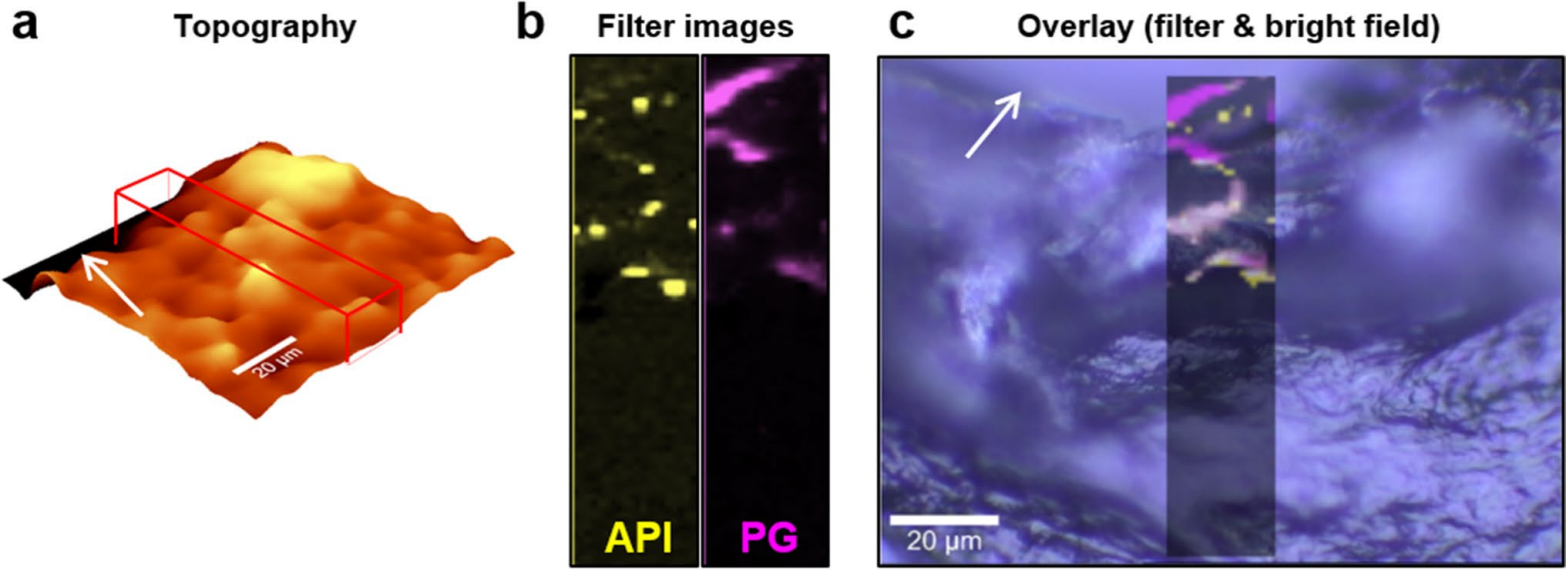
Combination of white light reflectance for topographic measurements and CRM on a skin sample dosed with an acyclovir/PG solution prior to cryosectioning. (**a**) Surface mapping by white light reflectance. (**b**) Single-Raman-peak filter images of acyclovir (yellow) and PG (pink) of the region marked by the red box in (**a**). (**c**) Combination of Raman filter images and bright field microscopy of the cryosection. The skin surface is indicated by the white arrows.

Although the non-invasive character of CRM is notable because of its potential to be developed for eventual clinical use with human subjects, the method is also highly compatible with destructive tissue preparation techniques to achieve a more comprehensive analysis of a sample. The freeze drying of biological tissues and their analysis by CRM has the added benefit of preserving the original state of the tissue while simultaneously intensifying Raman scattering by eliminating water from the sample (41). Freezing the sample also prevents further penetration of a semisolid formulation into the tissue, which could potentially confound the results in CRM studies with long acquisition times. While CRM can analyze an entire, intact freeze-dried skin sample, and thereby prevent any tissue damage, technical challenges related to increasing signal attenuation as a function of acquisition depth limit acquisition of suitable data deeper within the skin tissue. To address this issue, fresh tissue can be frozen in liquid nitrogen and cryosectioned; the individual cryosections can subsequently be analysed by CRM, circumventing some of the limitations of signal attenuation as a function of acquisition depth. When working with very thin cryosections (~ 20 µm), the acquisition of a topographic map of the (often rough) sample surface with white light reflectance is a necessary step to facilitate Raman measurements directly on the surface of the skin section, thereby ensuring good signal intensity. Figure 8 depicts an example of the combination of bright field microscopy, white light interferometry, and Raman microscopy carried out on a cryosection of skin that was previously incubated with a saturated solution of acyclovir in PG for 24 h.

The combination of white light reflectance measurements and confocal Raman microscopy allows for the mapping of the sample surface (Fig. 8(a)) and ensures better signal intensity by recording every Raman spectrum that is acquired in the scan area directly on the sample surface. Filter images, which show intensity distributions for a selected peak in the form of false color images, can be used to visualize the distribution of a chemical compound in the analyzed sample (Fig. 8(b)). By creating an overlay of the two filter images of PG (pink) and acyclovir (yellow) with the bright field microscopic image, penetration into the skin tissue can be visualized in a manner that preserves the context relative to the anatomical structures of the skin, and which can thereby provide even further insights into not only how compounds penetrate into the skin, but also how they distribute in skin tissues (Fig. 8(c)).

## Conclusion

This work illustrates the power and utility of Raman spectroscopy and microscopy as valuable techniques for the chemically-selective physicochemical and structural characterization of semisolid dosage forms. The chemically-selective nature of the method allowed for an in-depth investigation of different acyclovir creams, 5% products, comparing a set of reference products with each other and with ‘generic’ counterparts. Raman spectroscopy and microscopy techniques were able to characterize differences in the physicochemical and structural attributes of the products. The significance of this work is that differences in potentially critical physicochemical and structural quality attributes between reference and prospective generic products can be associated with potential failure modes for BE, and the results described here demonstrate that Raman spectroscopy and microscopy can provide detailed information about the components of a formulation, the arrangement of matter and structural features in the dosage form, the polymorphs of a substance that may be present in a topical product, and other attributes that can be used to directly compare the physicochemical and structural similarity of a prospective generic product to its reference product, thereby potentially mitigating the risk of specific failure modes for BE. In addition to characterizing and comparing product quality attributes, CRM was also demonstrated to be a useful tool for evaluating and comparing product performance, by characterizing the penetration kinetics of compounds from topical semisolid products into the upper layers of human skin. The correlation of cutaneous PK results characterized by CRM with corresponding IVPT results for the same products highlights the usefulness of CRM as a method for profiling the penetration and distribution of topically applied compounds into the skin. In addition, this work illustrated that CRM can simultaneously, and non-invasively, monitor the penetration and/or distribution of topical formulations into the skin, thereby providing a more comprehensive approach by which to compare topical products and to study the mechanisms of topical drug delivery. Although CRM has been implemented for the analysis of interactions of topical formulations with the human skin tissue in several studies, the here presented work not only evaluated the penetration and permeation abilities of commercial formulations but further describes a systematic evaluation of the topical products by CRM. The here presented study thereby provides a novel and promising approach by which to develop and evaluate prospective generic topical dermatological drug products.

## Abbreviations

API: Active pharmaceutical ingredient
BA: Bioavailability
BE: Bioequivalence
CCD: Charge-coupled device
CRM: Confocal Raman microscopy
HPLC: High performance liquid chromatography
IVPT: In vitro Permeation testing
PBS: Phosphate buffered saline
PK: Pharmacokinetics
PG: Propylene glycol
RLD: Reference listed drug
TGA: Thermogravimetric analysis

## References

[1] Williams AC, Barry BW. Penetration enhancers. Adv Drug Deliv Rev. 2004;56(5):603–18. [Journal Article] 10.1016/j.addr.2003.10.025 15019749

[2] Lopes LB, Garcia MT, Bentley MV. Chemical penetration enhancers. Ther Deliv. 2015;6(9):1053–61. [Journal Article] 10.4155/tde.15.61 26458111

[3] Yacobi A, Shah VP, Bashaw ED, Benfeldt E, Davit B, Ganes D, *et al*. Current challenges in bioequivalence, quality, and novel assessment technologies for topical products. Pharm Res. 2014;31(4):837–846. [Journal Article] 10.1007/s11095-013-1259-1 24395404

[4] Braddy AC, Davit BM, Stier EM, Conner DP. Survey of international regulatory bioequivalence recommendations for approval of generic topical dermatological drug products. AAPS J. 2015;17(1):121–33. [Journal Article] 10.1208/s12248-014-9679-3 PMC4287290 25344440

[5] Chen ML, Shah VP, Crommelin DJ, Shargel L, Bashaw D, Bhatti M, *et al*. Harmonization of Regulatory Approaches for Evaluating Therapeutic Equivalence and Interchangeability of Multisource Drug Products: Workshop Summary Report. AAPS J. 2011;13(4):556–64. [Journal Article] 10.1208/s12248-011-9294-5 PMC3231855 21845486

[6] Chang RK, Raw A, Lionberger R, Yu L. Generic development of topical dermatologic products: formulation development, process development, and testing of topical dermatologic products. AAPS J. 2013;15(1):41–52. [Journal Article] 10.1208/s12248-012-9411-0 PMC3535108 23054971

[7] Kryscio DR, Sathe PM, Lionberger R, Yu L, Bell MA, Jay M, *et al*. Spreadability Measurements to Assess Structural Equivalence (Q3) of Topical Formulations—A Technical Note. AAPS Pharm Sci Tech. 2008;9(1):84–6. [Journal Article] 10.1208/s12249-007-9009-5 PMC2976873 18446465

[8] Rowe RC, Bray D. Water distribution in creams prepared using cetostearyl alcohol and cetrimide. J Pharm Pharmacol. 1987;39(8):642–3. [Journal Article] 10.1111/j.2042-7158.1987.tb03443.x 2888856

[9] Ribeiro HM, Morais JA, Eccleston GM. Structure and rheology of semisolid o/w creams containing cetyl alcohol/non-ionic surfactant mixed emulsifier and different polymers. Int J Cosmet Sci. 2004;26(2):47–59. [Journal Article] 10.1111/j.0412-5463.2004.00190.x 18494913

[10] Bioavailability and Bioequivalence Requirements. Sect. Code of Federal Regulations, Title 21, Chapter I, Subchapter D, Part 320 (2021). [Federal Regulation]

[11] Bartosova L, Bajgar J. Transdermal drug delivery in vitro using diffusion cells. Curr Med Chem. 2012;19(27):4671–7. [Journal Article] 10.2174/092986712803306358 22934776

[12] Trottet L, Owen H, Holme P, Heylings J, Collin IP, Breen AP, *et al*. Are all aciclovir cream formulations bioequivalent? Int J Pharm. 2005;304(1–2):63–71. [Journal Article] 10.1016/j.ijpharm.2005.07.020 16139970

[13] Kraeling ME, Zhou W, Wang P, Ogunsola OA. In vitro skin penetration of acetyl hexapeptide-8 from a cosmetic formulation. Cutan Ocul Toxicol. 2015;34(1):46–52. [Journal Article] 10.3109/15569527.2014.894521 24754410

[14] Kezutyte T, Kornysova O, Maruska A, Briedis V. Assay of tolnaftate in human skin samples after in vitro penetration studies using high performance liquid chromatography. Acta Pol Pharm. 2010;67(4):327–34. [Journal Article] 20635527

[15] Guth K, Schafer-Korting M, Fabian E, Landsiedel R, van Ravenzwaay B. Suitability of skin integrity tests for dermal absorption studies in vitro. Toxicol In Vitro. 2015;29(1):113–23. [Journal Article] 10.1016/j.tiv.2014.09.007 25280455

[16] Seto JE, Polat BE, VanVeller B, Lopez RFV, Langer R, Blankschtein D. Fluorescent Penetration Enhancers for Transdermal Applications. J Control Release. 2012;158(1):85–92. [Journal Article] 10.1016/j.jconrel.2011.10.018 PMC3294199 22062691

[17] Volz P, Boreham A, Wolf A, Kim TY, Balke J, Frombach J, *et al*. Application of Single Molecule Fluorescence Microscopy to Characterize the Penetration of a Large Amphiphilic Molecule in the Stratum Corneum of Human Skin. Int J Mol Sci. 2015. p. 6960–77. [Journal Article] 10.3390/ijms16046960 PMC4424999 25826528

[18] Rancan F, Papakostas D, Hadam S, Hackbarth S, Delair T, Primard C, *et al*. Investigation of polylactic acid (PLA) nanoparticles as drug delivery systems for local dermatotherapy. Pharm Res. 2009;26(8):2027–36. [Journal Article] 10.1007/s11095-009-9919-x 19533305

[19] Alvarez-Roman R, Naik A, Kalia YN, Fessi H, Guy RH. Visualization of skin penetration using confocal laser scanning microscopy. Eur J Pharm Biopharm. 2004;58(2):301–16. [Journal Article] 10.1016/j.ejpb.2004.03.027 15296957

[20] Bernard S, Beyssac O, Benzerara K. Raman mapping using advanced line-scanning systems: geological applications. Appl Spectrosc. 2008;62(11):1180–8. [Journal Article] 10.1366/000370208786401581 19007458

[21] Carey PR. Raman crystallography and other biochemical applications of Raman microscopy. Annu Rev Phys Chem. 2006;57:527–54. [Journal Article] 10.1146/annurev.physchem.57.032905.104521 16599820

[22] Cantarero A. Raman Scattering Applied to Materials Science. Procedia Materials Sci. 2015;9:113–22. [Journal Article]

[23] Vankeirsbilck T, Vercauteren A, Baeyens W, Van der Weken G, Verpoort F, Vergote G, *et al*. Applications of Raman spectroscopy in pharmaceutical analysis. Trends Analyt Chem. 2002;21(12):869–77. [Journal Article]

[24] Paudel A, Raijada D, Rantanen J. Raman spectroscopy in pharmaceutical product design. Adv Drug Deliv Rev. 2015;89:3–20. [Journal Article] 10.1016/j.addr.2015.04.003 25868453

[25] Essendoubi M, Gobinet C, Reynaud R, Angiboust JF, Manfait M, Piot O. Human skin penetration of hyaluronic acid of different molecular weights as probed by Raman spectroscopy. Skin Res Technol. 2016;22(1):55–62. [Journal Article] 10.1111/srt.12228 25877232

[26] Liu Y, Lunter DJ. Profiling skin penetration using PEGylated emulsifiers as penetration enhancers via confocal Raman spectroscopy and fluorescence spectroscopy. Eur J Pharm Biopharm. 2021;166:1–9. [Journal Article] 10.1016/j.ejpb.2021.04.027 34082121

[27] Liu Y, Krombholz R, Lunter DJ. Critical parameters for accurate monitoring of caffeine penetration in porcine skin using confocal Raman spectroscopy. Int J Pharm. 2021;25;607:121055[Journal Article] 10.1016/j.ijpharm.2021.121055 34461169

[28] Lutker KM, Quinones R, Xu J, Ramamoorthy A, Matzger AJ. Polymorphs and hydrates of acyclovir. J Pharm Sci. 2011;100(3):949–63. [Journal Article] 10.1002/jps.22336 PMC3140643 21280051

[29] Information on Zovirax^®^ 5 % acyclovir cream (UK), via the electronic medicines compendium (emc); https://www.medicines.org.uk/emc/product/5468/smpc [Website]

[30] Patent information on Zovirax^®^ 5 % acyclovir cream (US), via the center for drug evaluation and research; https://www.accessdata.fda.gov/drugsatfda_docs/nda/2002/21-478_Zovirax_admindocs.pdf [Website]

[31] Package insert of Zovirax^®^ 5 % acyclovir cream (Austria), via Arzneispezialitätenregister, Bundesamt für Sicherheit im Gesundheitswesen, Österreich; https://aspregister.basg.gv.at/document/servlet?action=show&zulnr=1-18064&type=DOTC_FACH_INFO [Website]

[32] Package insert of Aciclovir 1A Pharma 5 % acyclovir cream (Austria), via Arzneispezialitätenregister, Bundesamt für Sicherheit im Gesundheitswesen, Österreich; https://aspregister.basg.gv.at/document/servlet?action=show&zulnr=1-22499&type=DOTC_FACH_INFO [Website]

[33] Kligman AM, Christophers E. Preparation of Isolated Sheets of Human Stratum Corneum. Arch Dermatol. 1963;88:702–5. [Journal Article] 10.1001/archderm.1963.01590240026005 14071437

[34] Namjoshi S, Caccetta R, Edwards J, Benson HA. Liquid chromatography assay for 5-aminolevulinic acid: application to in vitro assessment of skin penetration via Dermaportation. J Chromatogr B Analyt Technol Biomed Life Sci. 2007;852(1–2):49–55. [Journal Article] 10.1016/j.jchromb.2006.12.040 17236824

[35] Brittain HG. Polymorphism in pharmaceutical solids. New York: Marcel Dekker; 1999. [Book]

[36] Strachan CJ, Rades T, Gordon KC, Rantanen J. Raman spectroscopy for quantitative analysis of pharmaceutical solids. J Pharm Pharmacol. 2007;59(2):179–92. [Journal Article] 10.1211/jpp.59.2.0005 17270072

[37] Rawlings AV, Canestrari DA, Dobkowski B. Moisturizer technology versus clinical performance. Dermatol Ther. 2004;17 Suppl 1:49–56. [Journal Article] 10.1111/j.1396-0296.2004.04s1006.x 14728699

[38] Franz TJ, Lehman PA, Raney SG. Use of excised human skin to assess the bioequivalence of topical products. Skin Pharmacol Physiol. 2009;22(5):276–86. [Journal Article] 10.1159/000235828 PMC2790798 19707043

[39] Raney SG, Franz TJ, Lehman PA, Lionberger R, Chen ML. Pharmacokinetics-Based Approaches for Bioequivalence Evaluation of Topical Dermatological Drug Products. Clin Pharmacokinet. 2015;54(11):1095–106. [Journal Article] 10.1007/s40262-015-0292-0 26063051

[40] Franzen L, Windbergs M. Accessing Raman spectral variability in human stratum corneum for quantitative in vitro depth profiling. J. Raman Spectrosc., 45: 82–88. [Journal Article]

[41] Franzen L, Anderski J, Planz V, Kostka KH, Windbergs M. Combining confocal Raman microscopy and freeze-drying for quantification of substance penetration into human skin. Exp Dermatol. 2014;23(12):942–4. [Journal Article] 10.1111/exd.12542 25219950

